# Enhanced tactile acuity through mental states

**DOI:** 10.1038/srep13549

**Published:** 2015-08-27

**Authors:** Sebastian T. Philipp, Tobias Kalisch, Thomas Wachtler, Hubert R. Dinse

**Affiliations:** 1 Institute fur Neuroinformatics, Neural Plasticity Lab, Ruhr University, Bochum, Germany; 2 Department of Biology II, Ludwig-Maximilians-University Munich, Germany; 3 Clinic of Neurology, BG University Hospital Bergmannsheil, Ruhr-University, Bochum, Germany

## Abstract

Bodily training typically evokes behavioral and perceptual gains, enforcing neuroplastic processes and affecting neural representations. We investigated the effect on somatosensory perception of a three-day Zen meditation exercise, a purely mental intervention. Tactile spatial discrimination of the right index finger was persistently improved by only 6 hours of mental–sensory focusing on this finger, suggesting that intrinsic brain activity created by mental states can alter perception and behavior similarly to external stimulation.

The framework of neural plasticity captures the observation that training and practice and thus sensory stimulation drive reorganization of the brain, which in turn modifies perception and behavior^1^,^2^,^3^. However, recent experiments have demonstrated that mental imagery or feedback-induced modulation of brain activity can drive perceptual improvement in the absence of external stimulation or training^4^,^5^. These observations challenge our understanding of plasticity and learning processes. Here we went one step further and investigated psychophysically the perceptual learning effects of purely mental states on tactile acuity of the fingertips after a three-day meditative Zen retreat.

The two markers of tactile perceptual abilities that we assessed were spatial two-point discrimination (2pd) thresholds and localization performance. Both tests we applied to the tips of digits 2 and 3 (r2 and r3) of the right hand, and to test for possible transfer, also to digit 2 of the left hand (l2). The 2pd threshold is a reliable marker of tactile spatial discrimination abilities, and localization performance characterizes the ability to indicate the precise location of a low-threshold stimulus. It is known that perceptual learning induces a typical pattern of joint changes, where acuity improves, while localization becomes impaired^6^,^7^,^8^.

The Zen retreat was held in total silence for four days with long meditation periods (>8 hours per day). Participants were 20 experienced Zen scholars (3.9 ± 1.5 hours per week meditation practice; 11.6 ± 7.9 years of meditation experience) with ample experience in focused attention meditation (FAM) and open monitoring meditation (OMM). FAM is characterized by focusing sustained attention on a chosen object, and OMM is characterized by a nonspecific, non-reactive monitoring of the present content of experience without voluntarily focusing attention on an object^9^. During a three-day meditation period, participants in the sensory focusing group (n = 10; age: 49.9 ± 5.7; 4 women) were instructed to be completely aware of the spontaneously arising sensory perception in their right index finger (FAM) for a period of 2 hours per day while maintaining the normal meditative Zen practice (OMM) for the rest of the day (6 hours). On a fourth day, participants in the sensory focusing group practiced conventional OMM meditation for the whole day. Ten age-matched controls (age: 51.7 ± 4.2; 5 women) maintained their normal meditative practice (OMM) for the whole four days. Tactile performance of all participants was first measured before the retreat (pre-measure) and re-assessed after the three-day meditation period (post1 measure), and on day 4 (post2 measure). Because of the rigid meditation posture in Zen, the possibility of physical stimulation to the fingers during meditation can be excluded.

Compared to baseline in the same participants, after three days of meditation, in the sensory focusing group, 2pd performance improved significantly by an average of 17 ± 4% (SEM) for r2 (Wilcoxon’s test: p = 0.013) and by 12 ± 5% for r3 (p = 0.025), but not for l2 (7 ± 3%; p = 0.086; Figs 1 and 2). In the assessment on day 4, 2pd thresholds remained significantly lowered for r2 (22 ± 7%, p = 0.014) and r3 (15 ± 6%, p = 0.022) compared to baseline (pre), while l2 remained unchanged (5 ± 4%, p = 0.26). In contrast, localization performance as indicated by error rates remained unchanged on all fingers on day 3 [r2: 0.8 ± 5% (SEM), p = 0.92; r3: 5.5 ± 6%, p = 0.81; l2: 4 ± 5.3%, p = 0.57] and on day 4 (r2: 3.6 ± 3%, p = 0.33; r3: 0.9 ± 4%, p = 1.0; l2: 0.3 ± 5%, p = 0.94).

Participants in the control group showed no changes in 2pd thresholds on day 3 (r2: 3 ± 3%, p = 0.12; r3: 1 ± 3%, p = 0.45; l2: −6 ± 6%, p = 0.58) and day 4 (r2: −1 ± 3%, p = 0.92; r3: 2 ± 4%, p = 0.67; l2: 0.7 ± 4%, p = 0.96) and no changes in localization performance on day 3 (r2: 5.5 ± 5%, p = 0.33; r3: 3.8 ± 5%, p = 0.36; l2: 11.7 ± 4%, p = 0.13) or day 4 (r2: 2.5 ± 3%, p = 0.34; r3: 1 ± 4%, p = 0.51; l2: 6.2 ± 5%, p = 0.28).

Our data show that focused attention meditation on a particular body part—in this case the right index finger—significantly enhanced tactile acuity but not localization performance. In addition, open monitoring meditation resulted in no changes at all. These data indicate that merely being aware without external stimulation or training can drive highly specific changes in tactile perception. The observed amount of improvement in tactile acuity was comparable to or was even higher than that typically found following training^10^ or stimulation-induced learning^3^,^11^,^12^. By comparison, the superior acuity reported for musicians or the visually impaired is about 15 to 25% above that of typical individuals^12^. Thus, mental states maintained for several hours were associated with a substantial potential of inducing persistent perceptual alterations comparable in magnitude to those following long-term training or stimulation.

Recent experiments have shown that imagining the crucial, middle part of a visual bisection stimulus induces perceptual learning similar to perceptual improvement found for real stimulation conditions^4^. In those experiments, the imagined stimulus was part of a visual discrimination task. In contrast, in the sensory focusing group described here, participants were only asked to be aware of spontaneously arising perceptions in their right index finger. Thus, our findings indicate that merely being aware, without purposefully creating mental images of a specific stimulus configuration, can improve discrimination performance.

Prolonged meditation practice has significant effects on brain functioning^9^,^13^. For example, expert meditators have shown changes in attentional processing^14^. Other studies reported that meditators show increased cortical thickness in regions associated with attention, interoception, and awareness^15^, white matter changes^16^, and altered connectivity pattern^17^,^18^, suggestive of both short-term and long-lasting changes in brain activity. Moreover, there is agreement that meditation alters brain rhythms^13^. Eight weeks of mindfulness meditation training resulted in enhanced alpha power modulation. Interestingly, this modulation was specifically found for the low-alpha sub-band, which has been linked to attentional modulation^19^.

In our study we had used two different psychophysical tests targeting tactile discrimination and tactile localization abilities of the fingers. The rational for this was that previous training and stimulation experiments demonstrated a trade-off between 2pd and localization performance changes, indicating that both tasks are inherently interrelated^6^,^7^,^8^. Accordingly, when training or repetitive stimulation improve discrimination, localization performance is impaired. In contrast to this pattern, we here observed a novel pattern of changes, where discrimination improves, while localization remained unaffected. This specific pattern of tactile perceptual alterations is important in two respects: First, the lack of effects on localization behavior indicates that the gain in acuity is not simply the result of a nonspecific modulation of cortical processing as can be expected following meditation-induced enhanced attentional processing. In such a case, both tasks should have been similarly affected. Second, to then explain the lack of localization performance effects, we suggest that meditation induces changes in neural processing that must be different from those that mediate perceptual changes following training or repetitive stimulation^6^,^7^,^8^.

The primary somatosensory cortex (S1) contains a unilateral representation of the fingers whereas the second somatosensory cortex (S2) contains bilateral representations. Individual baseline acuity has been shown to correlate with the size of cortical S1 representations^20^, and with the resting BOLD fluctuation in S1^21^. Recent plasticity studies have shown that learning-induced tactile acuity improvement correlated with the amount of S1 enlargement of the finger representation^11^. Because changes in 2pd performance were found in the current work for the fingers r2 and r3 of the right hand, but not for the index finger of the left hand, the results presented here indicate an involvement of S1 in the plasticity process. Therefore, to explain the effects of FAM on sensory processing capabilities, we hypothesize that sensory focusing on the right index finger created neural activation in a region in S1, and that this self-generated activity has been instrumental for inducing plasticity processes, which in turn affected in a specific way sensory processing and thus tactile perception. In fact, recent imaging data have shown that sustained attention to spontaneous sensations of the thumb is sufficient to activate corresponding somatosensory and proprioceptive brain areas such as area 3a and 3b^22^. In addition, a wide-spread cortical-hippocampal-insular network is activated during spontaneous sensations of the thumb^23^. While we feel that our data cannot be explained by attentional modulation, we do assume that the sensory focusing evoked specific S1 activation which in turn induced specific plasticity processes. Because the control group (OMM) showed no perceptual changes, it appears conceivable that OMM alone without sensory focusing may be too nonspecific to induce changes of sensory processing.

Taken together, our findings indicate that the framework of neuroplastic processes induced by external training and stimulation needs to be extended to incorporate the observation that intrinsic brain activity created by mental states without external events can alter perception and behavior in similar ways.

## Methods

### Participants

In total, 20 subjects (9 female) with no previous history of psychological disorders or any known hand or head injuries were enrolled in the study. Subjects gave their written informed consent. The experimental protocol had been approved by the local ethics committee of the Ruhr-University Bochum and was performed in accordance with the Declaration of Helsinki.

### Assessment of two-point-discrimination thresholds

2pd thresholds were assessed using a custom-made device^11^,^12^. Stimuli consisted of seven pairs of brass needles with distances ranging from 0.7 to 2.5 mm in increments of 0.3 mm or from 1 to 4 mm in increments of 0.5 mm. In all cases, a single needle served as the control condition. Participants were not informed about the ratio of needle pairs and single needles. The stimuli were presented 10 times in randomized order resulting in 80 trials per session. Participants had to decide immediately after stimulus application if they had the sensation of 1 or 2 needles. All responses were plotted against needle distances, and data were fit by a sigmoidal function a *tanh(b (x − c)+1) via a least-squares method where a, b, and c were fitting parameters and x was the needle distance. The 2pd threshold was taken from the fit where 50% correct responses were reached. During all measurements, participants had to close their eyes. All participants had to accomplish one training session to become familiar with the testing procedure.

### Assessment of localization performance

Localization performance on the tip of the fingers was measured using a forced-choice paradigm in which participants had to report the absolute position on the tip of a finger where they perceived a touch sensation without visual inspection^8^,^11^. A small square (1 cm^2^) was printed on the skin of the fingertip, containing four quadrants of equal size (5 × 5 mm each) numbered 1 to 4. The center of each quadrant was touched in a pseudorandomized order 40 times with a von Frey filament (Marstocknervtest, Marburg, Germany) with a buckling force just above threshold. The threshold was determined before the localization assessment via a staircase procedure. Participants were instructed to report the number of the quadrant where they felt the sensation. To facilitate this procedure, participants were allowed to see a drawing of the fingertip with the four quadrants identified by numbers 1 to 4. Average localization performance is given for each finger by the rate of correct quadrant identifications.

## Additional Information

**How to cite this article**: Philipp, S. T. *et al*. Enhanced tactile acuity through mental states. *Sci. Rep*. **5**, 13549; doi: 10.1038/srep13549 (2015).

## Figures and Tables

**Figure 1:**
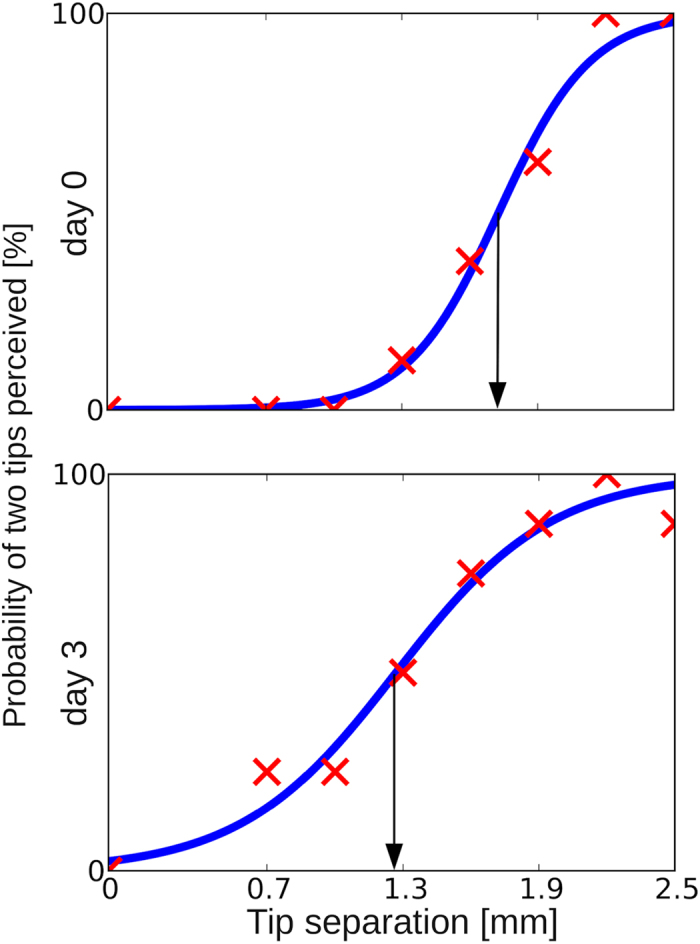
Psychometric 2pd curves of the right index finger of a single participant on day 0 (pre) and day 3 (post1). The probability that two tips were correctly perceived was plotted against needle distances (red crosses), which was fitted by a sigmoidal function (blue trace). The 2pd threshold was taken from the sigmoidal fit where 50% probability was reached. On day 0, the participant showed a 2pd threshold of 1.68 mm. On day 3, the threshold was lowered to 1.27 mm (an improvement in 2pd performance of 24%).

**Figure 2:**
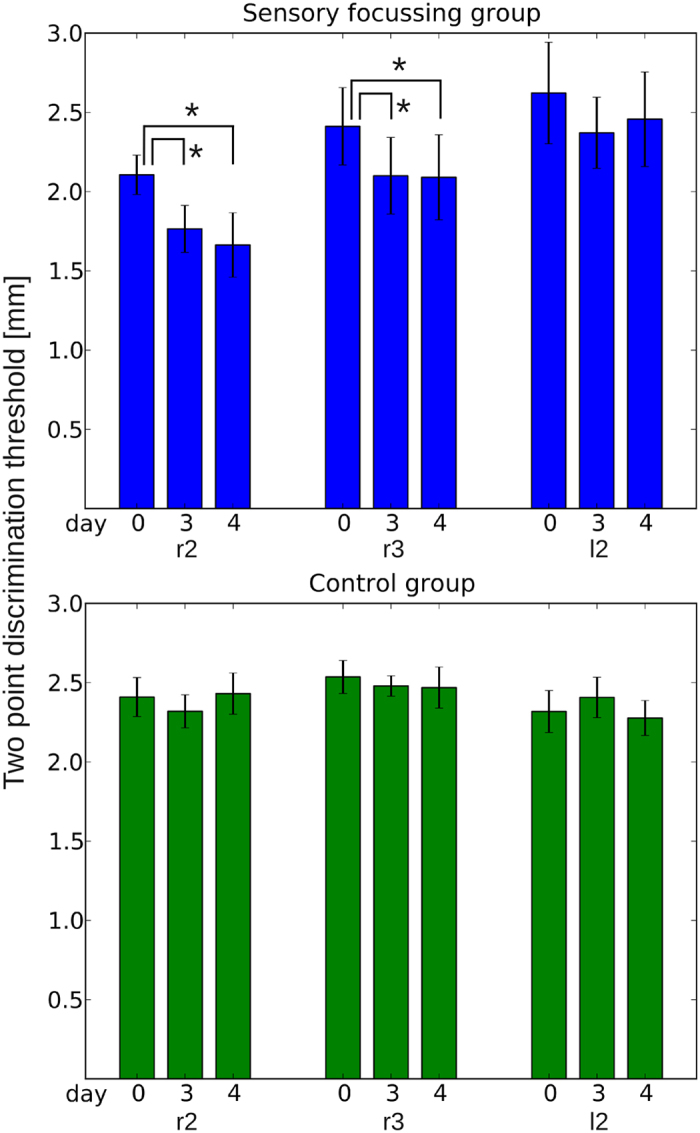
Changes in average two-point discrimination thresholds. For each finger and each group, average two-point discrimination thresholds and standard errors on day 0 (pre), day 3 (post1), and day 4 (post2) are shown. Compared to baseline, two-point discrimination thresholds in the sensory focusing group were lowered significantly for r2 on days 3 and 4 (Wilcoxon’s test p < 0.05). Controls showed no significant changes.
